# Magnetoencephalography and electroencephalography can both detect differences in cortical responses to vibrotactile stimuli in individuals on the autism spectrum

**DOI:** 10.3389/fpsyt.2022.902332

**Published:** 2022-08-05

**Authors:** Seppo P. Ahlfors, Steven Graham, Jussi Alho, Robert M. Joseph, Nicole M. McGuiggan, Zein Nayal, Matti S. Hämäläinen, Sheraz Khan, Tal Kenet

**Affiliations:** ^1^Athinoula A. Martinos Center for Biomedical Imaging, Massachusetts General Hospital, Charlestown, MA, United States; ^2^Department of Radiology, Massachusetts General Hospital and Harvard Medical School, Boston, MA, United States; ^3^Department of Neurology, Massachusetts General Hospital and Harvard Medical School, Boston, MA, United States; ^4^Department of Anatomy and Neurobiology, Boston University School of Medicine, Boston, MA, United States

**Keywords:** magnetoencephalography (MEG), EEG, somatosensory, vibrotactile, biomarker, inter-trial coherence (ITC), autism

## Abstract

Autism Spectrum (AS) is defined primarily by differences in social interactions, with impairments in sensory processing also characterizing the condition. In the search for neurophysiological biomarkers associated with traits relevant to the condition, focusing on sensory processing offers a path that is likely to be translatable across populations with different degrees of ability, as well as into animal models and across imaging modalities. In a prior study, a somatosensory neurophysiological signature of AS was identified using magnetoencephalography (MEG). Specifically, source estimation results showed differences between AS and neurotypically developing (NTD) subjects in the brain response to 25-Hz vibrotactile stimulation of the right fingertips, with lower inter-trial coherence (ITC) observed in the AS group. Here, we examined whether these group differences can be detected without source estimation using scalp electroencephalography (EEG), which is more commonly available in clinical settings than MEG, and therefore offers a greater potential for clinical translation. To that end, we recorded simultaneous whole-head MEG and EEG in 14 AS and 10 NTD subjects (age 15–28 years) using the same vibrotactile paradigm. Based on the scalp topographies, small sets of left hemisphere MEG and EEG sensors showing the maximum overall ITC were selected for group comparisons. Significant differences between the AS and NTD groups in ITC at 25 Hz as well as at 50 Hz were recorded in both MEG and EEG sensor data. For each measure, the mean ITC was lower in the AS than in the NTD group. EEG ITC values correlated with behaviorally assessed somatosensory sensation avoiding scores. The results show that information about ITC from MEG and EEG signals have substantial overlap, and thus EEG sensor-based ITC measures of the AS somatosensory processing biomarker previously identified using source localized MEG data have a potential to be developed into clinical use in AS, thanks to the higher accessibility to EEG in clinical settings.

## Introduction

Autism Spectrum (AS) is a defined primarily by differences in social interactions relative to neurotypical individuals, with differences in sensory processing also characterizing the condition (1). Differences in the cortical processing of sensory stimuli may provide a basis for developing neurophysiological biomarkers that can be associated with specific traits of AS, and thus facilitate the assessment of interventions targeting these domains (2). Of particular interest is the somatosensory domain, as many prior studies have identified tactile processing differences in AS. These include behavioral differences during somatosensory discrimination tasks, differences in evoked responses to somatosensory stimuli, differences in cortical functional connectivity associated with somatosensory cortices, and differences in metabolite levels associated with sensory processing traits (3–16).

Motivated by such findings, and in particular by findings of different behavioral responses to vibrotactile stimuli in AS (17), we previously showed that these vibrotactile-stimulation driven behavioral changes have a distinct associated neurophysiological signature, identified using magnetoencephalography (MEG) (18). More specifically, Khan et al. identified differences between AS and neurotypically developing (NTD) subjects in the somatosensory steady-state response to a 25-Hz vibrotactile pneumatic stimulus applied to the tips of the index and middle fingers. The response consisted of two components: a 25-Hz inter-trial coherence (ITC) component, as expected, and a 50-Hz ITC component which was not phase-locked to the 25-Hz component. Furthermore, the envelopes of the two components of the response were also uncorrelated. Additional analyses showed that the 50-Hz component is indeed not a harmonic, and were likely generated through local and long-range feedback mechanisms.

Group comparison analyses of these two components of the response showed that cortically-constrained MEG source estimates of the response showed lower ITC in the AS than the NTD group in the 50-Hz component in the primary somatosensory cortex (S1), with a large effect size, and higher ITC in the 25-Hz component in the secondary somatosensory cortex (S2), with a more moderate effect size. The simple and accessible design, using passive vibrotactile stimulation, makes it a compelling potential translational target for populations that are more deeply affected by AS, or to younger participants that would not be able to participate in more demanding paradigms. However, the MEG setup and the focus on data at the source level are not ideal for translation, as MEG resources are relatively scarce, and source space analyses typically require specialized skills. That said, electroencephalography (EEG) and MEG tend to capture similar information [see e.g., Ahlfors (19)], which led us to ask whether the same group difference could be captured by EEG as well. Because EEG is more available in clinical settings than MEG, it would be highly desirable in terms of clinical translation if the proposed trait specific biomarker candidates identified with MEG could be obtained using EEG, preferably using only a small number of electrodes and without the need for source estimation analyses.

More specifically, MEG and EEG source estimation (or “source localization”) procedures help to dissociate between contributions from multiple simultaneously active brain regions (20). However, source estimation imposes practical challenges by requiring a moderately high number of sensors (typically 30 +) and a conductivity model for the head (typically a spherical model or information from anatomical MRI is used). When pursuing domain specific neurophysiological biomarkers, one potential approach is to first apply advanced source estimation methods to large-array multi-channel MEG and/or EEG data to identify the brain regions contributing to the measure of interest. Once these patterns of source activity have been determined, they can be used to guide the optimal placement of a small number of sensors for detecting directly the effects of interest, without further source estimation analyses. For example, if MEG source estimates reveal the locations of some specific somatosensory evoked responses to be at S1 and S2 cortical areas (21), this information can be used, by solving the so called forward problem (22), to predict what the signals would be in EEG electrodes at different scalp locations.

The translation from MEG to EEG is in theory feasible because MEG and EEG signals have a common physiological origin, namely, electrical currents associated with neural activity. The sources of the MEG and EEG signals are described in terms of active “primary” currents, mostly corresponding to post-synaptic dendritic currents in cortical pyramidal neurons (23). Thus, similar effects of interest, including differences between AS and NTD groups in the response to tactile stimulation, are expected to be found in MEG and EEG data. However, the MEG and EEG have different patterns of sensitivity to the location, orientation, and extent of the source currents (24–26), and therefore the same effects may not necessarily be found. Furthermore, MEG and EEG are also differently affected by the conductivity properties of the head, particularly the skull (27). Some activity may be detectable only in MEG or only in EEG, depending on the available SNR (26, 28, 29). Thus, the question of whether specific effects identified using MEG source estimates can also be observed in EEG scalp data needs, in general, to be to be addressed empirically for each paradigm being studied.

Here, we examined whether the group differences observed in MEG source estimates reported by Khan et al. (18) using this simple, translational vibrotactile paradigm, can be detected using scalp EEG without source estimation. To that end, we recorded simultaneous 306-channel MEG and 70-channel EEG in 14 AS and 10 NTD subjects (age 15–28 years) using the same vibrotactile paradigm.

## Materials and methods

### Participants

Fourteen AS participants and ten NTD control participants were studied. No participants were excluded, as data from all participants was of sufficiently high quality. Parents of participants ages 15–17 years, and all adult participants ages 18 and up, provided informed consent according to protocols approved by the Massachusetts General Hospital and Institutional Review Board (IRB). Participants ages 14–17 provided assent (verbal or written) in addition to parental consent.

Phenotypic data collected from all participants are summarized in Table 1. The age range was 15–28 and 15–23 years in the AS and NTD groups, respectively. The overall mean age of was 20.6 and the median age 20.4. All participants were right-handed, with the exception of two AS individuals who were left-handed and one AS individual who was ambidextrous, as determined with information collected using the Dean Questionnaire (30). Participants with AS had a clinical diagnosis of AS and met on the Autism Diagnosis Observation Schedule, Version 2 (ADOS-2) (31–33) administered by a trained research personnel with established inter-rater reliability. The Social Communication Questionnaire, Lifetime Version (SCQ Lifetime) (34) and Social Responsiveness Scale (SRS) (35) were administered to further confirm AS in AS participants and to rule out AS in NTD participants. AS participants who did not meet a cutoff of > 15 on the SCQ, a Total T score of > 59 on the SRS or had a borderline score on the ADOS-2 were further evaluated by expert clinician and co-author (RMJ) to confirm the AS diagnosis. Individuals with autism-related medical conditions (e.g., Fragile-X syndrome, tuberous sclerosis) and other known risk factors (e.g., gestation < 36 weeks) were excluded from the study. All NTD participants were below the threshold on the SCQ Lifetime questionnaire. Parent reports and Self-reports were administered to confirm that participants were free of any neurological or psychiatric conditions and substance use in the past 6 months. Verbal IQ (VIQ) and non-verbal IQ (NVIQ) were assessed using the Kaufman Brief Intelligence Test–II (36). Note that the group difference in VIQ is expected because VIQ is considered part of the AS phenotype (37). To assess the extent of sensory symptoms, we collected the Adults/Adolescents Sensory Profile (AASP) (38), a self-report questionnaire that quantifies sensory symptoms. We specifically focused on the scores from the somatosensory section of the AASP (AASP_*Som*_). Data on the AASP were collected for all but one NTD participant.

**TABLE 1:** Characterization of the participants.

	ASD: *n* = 14, 4 females	TD: *n* = 10, 2 females	*p*
Age	20.5 (1.15)	20.8 (0.87)	0.83
NVIQ	99 (4.9)	119 (3.8)	0.006
VIQ	94.4 (4)	118.4 (4.5)	0.001
ADOS_TOT_	11.5 (0.9)	−	−
SCQ_Lifetime_	16.6 (2.1)	3.3 (0.8)	0.003
SRS_TOT_	67.8 (2.4)	43.3 (1.5)	<10^–6^
AASP_TOT_	41.9 (3.25)	32.8 (2.2)	0.13
AASP_Som_	7.7 (0.65)	2.9 (1.4)	0.003

Mean (and SE) value are shown, with p-values from t-tests between the groups.

NVIQ, non-verbal IQ; VIQ, verbal IQ; ADOS_TOT_, autism diagnosis observation schedule total score (social affect + restricted and repetitive behavior); SCQ_Lifetime_, social communication questionnaire, lifetime version total score; SRS_TOT_, social responsiveness scale total T-score; AASP_TOT_, adult-adolescent sensory profile total score; AASP_Som_, adult-adolescent sensory profile touch processing subtotal score.

### Tactile stimulation

Vibrotactile stimulation consisted of pulses applied to the right index finger at 25 Hz using a custom made pneumatic tactile stimulator with latex tactor tips, based on a published design (39). The stimulator was the same as was used previously (18). The duration of each stimulus train was 1,000 ms (versus 500 ms previously) and the interstimulus interval (ISI) varied between 2.75 and 3.25 s (versus 1–2 s previously). These changes were made with the hope that the longer duration and ISI would increase the robustness of the putatively less selective sensor-space data. A total of 65 stimulus trains were presented to each subject. The stimuli were generated outside the magnetically shielded room and delivered to the subject *via* a long tube; the subjects could not hear sounds associated with the stimulation. The stimuli were presented while participants were watching a silent video of their choice. Participants were instructed to not pay attention to the stimulation and not move their hands, which we ensured were resting comfortably on a pillow. The sequence of stimuli was controlled using the MATLAB Psychophysics Toolbox.^1^

### Magnetoencephalography and electroencephalography data acquisition

MEG and EEG data were simultaneously acquired using a Neuromag Vectorview system (MEGIN Oy, Espoo, Finland) in a magnetically shielded room (Imedco, Olten, Switzerland). The system included 306 MEG channels (204 orthogonal planar gradiometers and 102 magnetometers), and a 70-channel EEG cap with nose reference and two electrooculogram channels. The data were band-pass filtered between 0.1 and 330 Hz prior to sampling at 1,000 Hz. The position of the head was continuously recorded during the data acquisition using four head position indicator (HPI) coils attached to the scalp. The locations of the HPI coils, three anatomical landmarks (nasion and auricular points), all EEG electrodes, and multiple additional scalp surface points were measured using a Fastrak digitizer (Polhemus).

### Magnetoencephalography and electroencephalography data preprocessing

Channels with excessive noise were identified with the Autoreject software using the algorithm’s default parameters (40), and excluded from further analyses. To compensate for head movements during the recording, and to reduce signals not originating in the brain, temporal Signal Space Separation (tSSS) (41) as implemented in MNE-Python (42, 43) was applied to the MEG data. MEG and EEG data were then band-pass filtered between 0.1 and 144 Hz, as well as notch-filtered at 60 and 120 Hz to remove power line noise. Cardiac and ocular artifacts were removed using the Signal Space Projection (SSP) method (44). The data were epoched into single trials lasting 3 s, from 1,000 ms prior to stimulus onset to 2,000 ms after. Both before and after preprocessing, the overall data quality for two groups were similar in terms of the number of trials per participant, the number of removed epochs, and the number of good EEG and MEG channels.

### Inter-trial coherence

To compute the ITC for the somatosensory event-related responses, the epoched sensor data were transformed to the time-frequency domain using Morlet wavelet decomposition in the frequency range of 20–80 Hz. The number of cycles for each wavelet was 1/3 of the frequency value. The time step was 3.03 ms and the frequency step was 1 Hz. ITC is a measure of consistency of the phase of oscillatory responses across trials (45); ITC values range from 0 to 1, with one corresponding to an identical phase in all trials.

### Statistical analysis

Measures for the somatosensory event-related response components were obtained by averaging the ITC values across ± 1-Hz frequency bins at 25 and 50 Hz, and over the 250 to 1,000-ms post-stimulus-onset time window, i.e., the steady-state component of the response. Note that the prior study (18) also focused on the steady-state component starting at 250 ms post stimulus onset. Because the ITC values are always positive and not expected to be normally distributed, we used non-parametric tests. Group differences in these measures were evaluated using 2-sided Wilcoxon rank-sum test. Effect sizes were calculated using Hedges’s *g*, which provides a correction for small sample sizes (46). Correlations between the ITC values obtained from MEG vs. EEG data, as well as their correlations with behavioral measures were evaluated using Spearman rank-order test.

We compared the groups along two different dimensions of the data: response frequency (25 and 50 Hz) and recording modality (MEG and EEG). Because all these measures are correlated, correction for multiple (here four) comparisons using the Bonferroni correction, which assumes independence, is too conservative. Furthermore, the premise for the comparisons was to reproduce specific *a priori* hypothesized group differences observed in the previous study (18). Therefore, we chose to report unadjusted *p*-values for the results of the group comparisons.

To further examine the relationship between the MEG and EEG ITC measures, a population-wide (AS and NTD combined) multimodal analysis was performed. A correlation matrix was computed using Pearson’s correlation. The effective rank of the MEG and EEG ITC measures at 25 and 50 Hz across subjects was evaluated using Principal Component Analysis (PCA).

## Results

**FIGURE 1:**
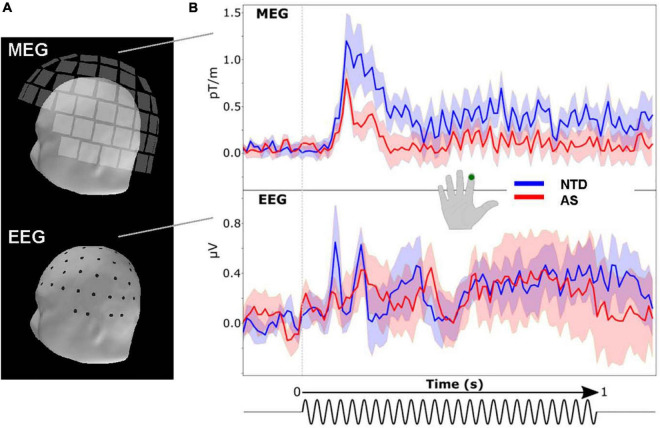
Averaged evoked response to 25-Hz vibrotactile stimulation of the index finger of the right hand. **(A)** Layout of the MEG (*top*) and EEG (*bottom*) sensor arrays. The squares represent MEG triple-sensor units (one magnetometer and two planar gradiometers) and the dots EEG scalp electrodes. **(B)** Waveforms from one representative left-hemisphere MEG planar gradiometer and EEG electrode were averaged over the subjects in the NTD (*blue*) and AS (*red*) groups; shading shows standard error across subjects. The time course of the stimulus is shown at the bottom.

Grand average (i.e., averaged over trials and subjects) somatosensory evoked responses to the vibrotactile stimulation in one representative MEG and one representative EEG sensor are shown in Figure 1. An oscillatory response at the 25-Hz stimulation frequency is visible in both MEG and EEG. Prominent transient deflections after the onset of the vibrotactile stimulus train are also evident.

**FIGURE 2:**
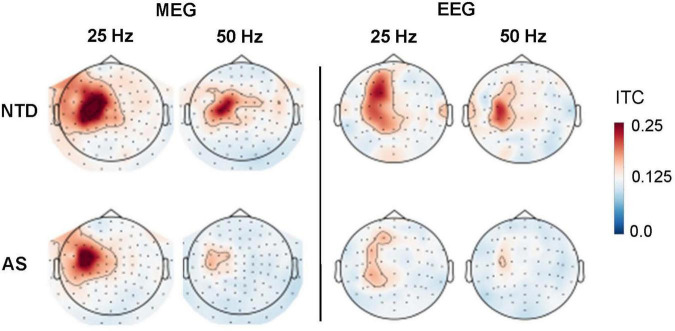
Topographic maps of the Inter-trial coherence (ITC) at 25 and 50 Hz in MEG gradiometers (*left*) and EEG sensors (*right*) for NTD (*top*) and AS (*bottom*) groups. The ITC values were averaged over the 250–1,000 ms post-stimulus-onset time window. The small dots indicate sensor locations.

The spatial distributions of the ITC for the 25 and 50-Hz response components, averaged over the 250–1,000 ms time window are shown in Figure 2. The largest ITC values were obtained in left frontoparietal MEG (planar gradiometer) sensors and in left frontoparietal EEG sensors, consistent with a source in the primary somatosensory cortex contralateral to stimulated finger. These topographic maps indicate that ITC was detectable at both 25 and 50 Hz in the group-averaged MEG and EEG sensor data. The maps suggested weaker ITC in the AS than in the NTD group for both the 25 and 50-Hz components of the response.

**FIGURE 3:**
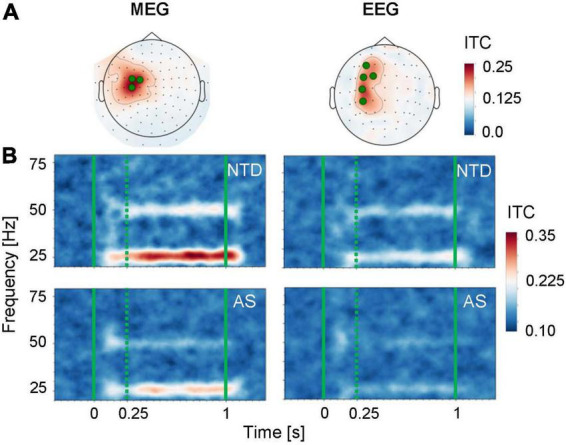
Time-frequency representation of the ITC for the responses to the 25-Hz tactile stimulation. **(A)** Topographic maps of the ITC values were averaged over all subjects (NTD and AS combined) within the 25 and 50-Hz frequency bins and the 250–1,000 ms time window. The green dots indicate frontocentral MEG (*left*) and EEG (*right*) sensors that were selected for all subsequent analyses. **(B)** Time-frequency maps of the ITC for the NTD (*top*) and AS (*bottom*) groups, averaged over the selected sensors. The solid green vertical lines indicate the onset and offset times of the stimulus, and the green dotted line indicates the beginning of the time window used for the analysis of the steady state component of the response.

For statistical analyses, we selected a small set of sensors (3 MEG planar gradiometers and 5 EEG electrodes) that showed large ITC values in the 250–1,000 ms time window in the population-wide averages (Figure 3A). Note that the topographic maps are consistent with tangential source currents at or near the S1 cortex. For a tangential source, the MEG planar gradiometer sensors show the largest signals right above the source (47), whereas the EEG signals are largest on both sides of the source, resulting here in anterior and posterior maxima of the ITC. Time-frequency representations of the ITC, averaged over the selected sets of sensors, are shown in Figure 3B. The NTD group showed a prominent ITC at both 25 and 50 Hz in response to the 25-Hz tactile stimulus in both MEG and EEG, and the responses were weaker in both modalities in the AS group than in the NTD group.

**FIGURE 4:**
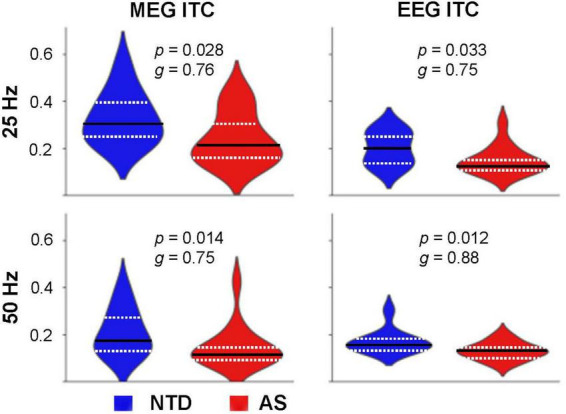
Distribution of the ITC values among the subjects in the NTD (*blue*) and AS (*red*) groups. The ITC values were averaged over the 250–1,000 ms time window and over the selected sensors. The dashed white lines indicate the first and third quartiles, the solid black line indicates the second quartile (median). *p*-values (2-sided Wilcoxon rank-sum test, uncorrected) and effect sizes (Hedges’s *g*) for the group differences are indicated for each case.

When averaging across the entire steady-state time window, 250–1,000 ms, the ITC values were significantly lower in the AS than in the NTD group for both modalities (Figure 4). For MEG 25 Hz: *p* = 0.028 (2-sided Wilcoxon rank sum test, uncorrected), Hedges’s *g* = 0.76 (effect size); MEG 50 Hz: *p* = 0.014, *g* = 0.75; EEG 25 Hz: *p* = 0.033, *g* = 0.75, EEG 50 Hz: *p* = 0.012, *g* = 0.88. Because the groups differed significantly on NVIQ, we then tested whether NVIQ values correlated with ITC values. No significant correlations between NVIQ and any of the ITC values were found in either group.

**FIGURE 5:**
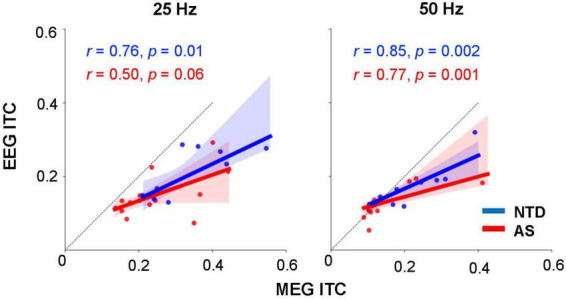
Relationship between the MEG and EEG ITC measures in individual subjects. Regression lines are shown separately for the two subject groups; shading indicates the 95% confidence interval. The *p*-values for the correlation coefficients (*r*) were computed using Spearman rank-order test, uncorrected. The dotted line with slope = 1 is shown for reference.

The MEG and EEG ITC measures in individual subjects were significantly correlated for both subject groups (Figure 5): for the NTD group, *r* = 0.76, *p* = 0.01 (25-Hz response, Spearman rank-order test, uncorrected) and *r* = 0.85, *p* = 0.02 (50-Hz); for the AS group, *r* = 0.50, *p* = 0.06 (25-Hz) and *r* = 0.77, *p* = 0.001 (50-Hz). Population wide, the correlations were: *r* = 0.67, *p* = 0.0004 (25-Hz) and *r* = 0.86, *p* < 10^–7^ (50-Hz).

**FIGURE 6:**
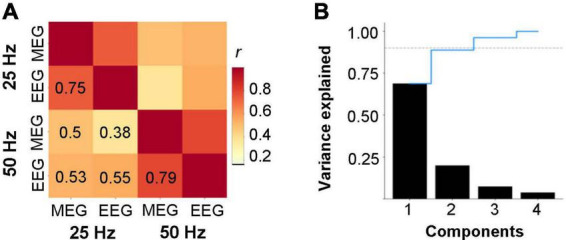
Population-wide properties of the ITC measures among the two modalities (MEG and EEG) and two response components (25 and 50 Hz). **(A)** Correlation matrix depicting the relationships between each modality’s and response frequency’s ITC measures. **(B)** Percentage of variance explained by each principal component. The dashed line shows the cumulative variance ratio explained. The dotted horizontal line indicates 0.9 level of variance explained, for reference.

Results of population-wide analysis of the relationship between the MEG and EEG ITC measures are shown in Figure 6A. The correlation matrix revealed that all four measures (MEG 25, EEG 25, MEG 50, and EEG 50 Hz) were correlated. The correlations were highest between the modalities (MEG and EEG) (0.75 and 0.79 for 25 and 50-Hz response, respectively). In other words, the correlations between the values of the same measure obtained using two different modalities (EEG or MEG) were higher than the correlations between the two components of the response (25 and 50 Hz) obtained within a single modality. The PCA indicated that two principal components can explain almost 90% of the variance (Figure 6B). Taken together, the correlation matrix and PCA suggest that the 25 and 50-Hz response components provide substantial complementary information, whereas MEG and EEG provide similar, overlapping information about each component.

**FIGURE 7:**
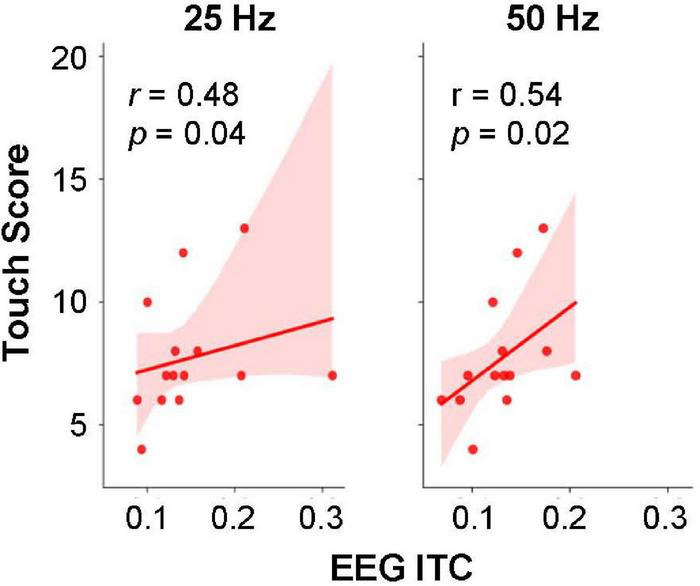
Relationship between EEG ITC values at 25 and 50 Hz and the Touch Processing–Sensation Avoiding section of the Adult Adolescent Sensory Profile (AASP_*Som*_) for subjects in the ASD group. The *p*-values for the correlation coefficients (*r*) were computed using Spearman rank-order test, uncorrected. Shading indicates 95% confidence interval.

Lastly, in Khan et al. (18) the ITC measures correlated with somatosensory processing measures obtained behaviorally. To examine whether the neural measures were related to sensory processing differences also in this smaller AS sample, the ITC values were correlated with behavioral Sensory Processing Scores of the AS participants, and specifically with each of the subscores with the “Touch” domain: “Low Registration,” “Sensation Seeking,” “Sensation Sensitivity,” and “Sensation Avoiding.” The highest correlations were found between EEG ITC (both 25 and 50-Hz) response and the Adult Adolescent Sensory Profile (AASP) Touch Processing subsection scores, as shown in Figure 7, with *p* = 0.04 for the 25-Hz ITC, and *p* = 0.02 for 50-Hz ITC, which however, would not survive a correction for multiple comparisons.

## Discussion

Differences between AS and NTD groups in steady-state somatosensory evoked responses were detected in both EEG and MEG sensor data. More specifically, lower ITC values in the AS than in the NTD group were found for both the 25-Hz response component as well as for the 50-Hz response component, at twice the stimulation frequency. Thus, in our small set of subjects, we detected similar group differences in the 50-Hz component of the response, using the same vibrotactile paradigm, as those in the MEG source estimation study of Khan et al. (18). These results confirm our hypothesis that this potential biomarke, targeted at somatosensory processing differences in AS and identified initially using source-localized MEG signal, is also identifiable using the more translational EEG sensor-space signal.

Indeed, the ITC measures obtained with MEG and EEG sensor data were notably correlated, and analysis of the relationship between the MEG and EEG measures suggested that the two response components, 25-Hz ITC and 50-Hz ITC, may provide more complementary information than the use of the two recording modalities do. This is not unexpected, considering the common physiological origin of the MEG and EEG signals, yet at the same time this could not be assumed to be the case without verification, given the known differences in MEG and EEG signals as well.

There were some notable differences between the present results and the prior study. Khan et al. (18) found lower source space (somatosensory areas S1 and S2) ITC in AS relative to NTD subjects only in the 50-Hz component of the response, whereas in the present sensor-based study lower ITC values were found in AS in both the 25-Hz and the 50-Hz components. In fact, in the prior study, the 25-Hz component of the response was larger in the AS group relative to the NTD group. These differences may have emerged due to differences in sample size and heterogeneity in participant’s characteristics among the studies; however, a more likely possibility is that they emerged due to the differences inherent in sensor-space versus source-space analyses. Source estimation, which helps to dissociate signals from multiple brain locations, was not attempted in the present study because that would not be feasible using clinical EEG with only a few electrodes. In the prior study, the group differences in the 25-Hz ITC were location dependent: the larger 25-Hz ITC in the AS group was only manifested in area S2, whereas the lower the 50-Hz response was found in both S1 and S2 (18). The discrepancy between the 25-Hz ITC results is therefore likely due to the relative contribution of S2 sources to surface EEG and MEG sensors being overwhelmed by contributions from the much larger response from S1. Indeed, the topographic maps for both MEG and EEG data in the present study were consistent with S1 sources, and the results were likely driven mostly by signals from S1 and neighboring areas.

Despite the differences between the two studies at the 25-Hz ITC responses, the observed group differences in 50-Hz ITC are sufficient for considering future potential clinical applications, and the advantages of the potential for clinical translation may outweigh the greater precision possible with source localization. Indeed, it is encouraging that the sensor EEG data showed group differences consistent with the source space analyses of S1 in the prior study, even if not every detail or nuance of the original study were captured. This is because the most robust results would still be captured by EEG, while higher accessibility raises the potential of clinical usefulness, for instance to objectively assess the extent of somatosensory processing differences in AS individuals and to customize treatment plans accordingly. Another potential avenue would be to then use the same paradigm as an intervention biomarker, to assess whether interventions aimed at easing sensory processing burdens in AS were, in fact, effective at the neurophysiological level.

Also encouraging is the indication of a potential correlation of the ITC values obtained using EEG with the sensory profile as assessed behaviorally. The *p*-values observed in this study would not survive a correction for multiple comparisons; however, because the different sensory processing scores are not independent and the MEG and EEG values are also not independent, the extent to which these *p*-values need to be corrected is not clear. Furthermore, the observed correlations are in line with the results of our prior study. Thus, although these correlations in and of themselves are not significant after correction, it is nonetheless encouraging that even with a small sample size, there is a trend in the direction of our prior result, supporting a correlation between EEG responses and behaviorally assessed somatosensory processing measures, as expected.

Some limitations of the study need noting. In particular, while the results are consistent with prior findings of EEG-measured responses to vibrotactile stimulation in a neurotypical population (48), and largely replicates a prior result (18), the relatively small sample size remains a limitation. For one, the small ratio of females to males precludes the analysis of potential gender-driven differences. Furthermore, although we hypothesize that the reason for only replicating the results for the 50-Hz component of the response is the lack of source localization and consequently the relative discounting of S2 contributions, a larger sample size might result in convergence with the prior study for the 25-Hz component of the response as well. It is also well known that the manifestation of sensory processing differences in AS is highly heterogenous, and therefore it is possible that the differences in the results are due to this heterogeneity between the sample of the prior study and the smaller sample size of the present study.

In conclusion, this study was motivated by the findings that somatosensory processing differences are common in AS (17, 49, 50), and focusing on biomarkers of sensory processing offers a path that is more likely to be translatable into animal models and across imaging modalities. The potential impact of this result is further augmented by the fact that the paradigm is simple and passive, and thus easily translatable to different age groups starting as soon as the steady state responses begins to stabilize in early childhood (51), as well as to AS populations with a broad range of abilities. The results also support a more general approach of first identifying target measures for group differences using MEG (or EEG) source localized data, and then using the results to translate recording guidelines and analysis of data to a setup that is based on just a handful of EEG channels.

## Data availability statement

The raw data supporting the conclusions of this article will be made available by the authors, without undue reservation.

## Ethics statement

The studies involving human participants were reviewed and approved by Massachusetts General Hospital Institutional Review Board. Written informed consent to participate in this study was provided by the participants’ legal guardian/next of kin.

## Author contributions

SK and TK designed the research. SA, SG, JA, NM, ZN, and TK collected the data. NM performed the behavioral assessments. RJ supervised the assessments and made final diagnosis decisions as applicable. SA, SG, MH, SK, and TK analyzed the data. SA, SG, SK, and TK wrote the manuscript. All authors contributed to the scientific discussion and approved the submitted version.

## Footnotes

1
www.psychtoolbox.org
